# OMIP‐086: Full spectrum flow cytometry for high‐dimensional immunophenotyping of mouse innate lymphoid cells

**DOI:** 10.1002/cyto.a.24702

**Published:** 2022-11-04

**Authors:** Kyle T. Mincham, Robert J. Snelgrove

**Affiliations:** ^1^ National Heart and Lung Institute Imperial College London London UK

**Keywords:** full spectrum flow cytometry, high‐dimensional flow cytometry, innate lymphoid cells, lung, small intestine lamina propria

## Abstract

This 25‐parameter, 22‐color full spectrum flow cytometry panel was designed and optimized for the comprehensive enumeration and functional characterization of innate lymphoid cell (ILC) subsets in mouse tissues. The panel presented here allows the discrimination of ILC progenitors (ILCP), ILC1, ILC2, NCR^+^ ILC3, NCR^−^ ILC3, CCR6^+^ lymphoid tissue‐inducer (LTi)‐like ILC3 and mature natural killer (NK) cell populations. Further characterization of ILC and NK cell functional profiles in response to stimulation is provided by the inclusion of subset‐specific cytokine markers, and proliferation markers. Development and optimization of this panel was performed on freshly isolated cells from adult BALB/c lungs and small intestine lamina propria, and ex vivo stimulation with phorbol 12‐myrisate 13‐acetate, ionomycin, and pro‐ILC activating cytokines.

## 1 PURPOSE AND APPROPRIATE SAMPLE TYPES

This 25‐parameter, 22‐color full spectrum flow cytometry panel was designed and optimized for the comprehensive enumeration and functional characterization of innate lymphoid cell (ILC) subsets in mouse tissues (Table 1). The panel presented here allows the discrimination of ILC progenitors (ILCP), ILC1, ILC2, NCR^+^ ILC3, NCR^−^ ILC3, CCR6^+^ lymphoid tissue‐inducer (LTi)‐like ILC3 and mature natural killer (NK) cell populations. Further characterization of ILC and NK cell functional profiles in response to stimulation is provided by the inclusion of subset‐specific cytokine markers, and proliferation markers. Development and optimization of this panel was performed on freshly isolated cells from adult BALB/c lungs and small intestine lamina propria, and ex vivo stimulation with phorbol 12‐myrisate 13‐acetate, ionomycin, and pro‐ILC activating cytokines.

**TABLE 1:** Summary table

Purpose	Deep immunophenotyping and functional assessment of ILC subsets
Species	Mouse
Cell types	Innate lymphoid cells
Cross‐reference	N/A

Abbreviation: ILC, innate lymphoid cell.

## 2 BACKGROUND

ILCs are a unique subset of innate effector cells enriched at mucosal surfaces, with diverse roles in host defense, tissue remodeling and repair, inflammation, and metabolic homeostasis [1]. Despite lacking rearranged antigen receptors, ILCs display remarkable homology with conventional T helper (Th) type 1 (Th1), Th2, and Th17 cells in regards to phenotype and function, and thus are similarly classified into ILC1, ILC2, and ILC3 subsets [2]. Moreover, bona fide ILC subsets have now been expanded to include both cytotoxic NK and lymphoid tissue‐inducer (LTi) cells, which are phenotypically and functionally similar to ILC1 and ILC3 respectively, yet exhibit distinct developmental trajectories [3]. However, it is increasingly recognized that ILC subsets are not fixed, and that these cells can exhibit significant plasticity depending on the local inflammatory milieu [4, 5, 6, 7, 8]. As such, ILCs demonstrate intra‐subset phenotypic heterogeneity depending on their specific microenvironment [9, 10], and deep immunophenotyping therefore requires a comprehensive array of phenotypic and functional markers to accurately capture this biological variation.

Although crucial in multiple biological settings, ILCs constitute a relatively minor population within both mouse and human lymphoid and non‐lymphoid tissues and blood, comprising 1%–5% of CD45^+^ leukocytes [5, 11, 12, 13, 14, 15]. Due to this inherent scarcity, acquiring as much information as possible on a single‐cell level is of upmost importance for accurate ILC discrimination. High resolution ILC characterization is further convoluted in tissues such as the lung and small intestine, where the intrinsically autofluorescent nature of the samples results in a heightened background noise‐to‐signal ratio. With this in mind, cellular characterization via full spectrum flow cytometry provides a technological advancement given its high parameter capabilities [16] combined with the capacity to extract cellular autofluorescence profiles, thus improving discrimination of rare populations compared to conventional flow cytometry [17]. As such, the full spectrum panel described herein enables the detailed enumeration and functional assessment of all ILC subsets identified within mouse tissues, with a specific focus on the characterization of lung and small intestine lamina propria (siLP) populations given the divergent repertoire of resident ILC subsets localized to these tissue compartments.

ILCs arise from common lymphoid progenitors within the bone marrow (BM), which through a series of intermediate commitment stages give rise to ILC progenitors (ILCP). While initially believed to be restricted within the BM, ILCPs are now recognized to exist within circulation and as tissue‐resident populations at distal sites in both neonates and adults, including the lungs, skin, and secondary lymphoid tissues [18, 19, 20, 21]. During early development, expression of the transcription factor promyelocytic leukemia zinc finger (PLZF; encoded by *Zbtb16*) dictates the bifurcation of innate lymphoid progenitors, whereby PLZF^+^ ILCPs are restricted to the generation of ILC1, ILC2, and ILC3, but not LTi or NK cells [22, 23]. In addition to PLZF, ILCPs display high‐level expression of *Il18r1*, and thus a requirement for IL‐18 signaling through IL‐18Rα for proliferation and differentiation [19]. Importantly, both ILCPs and mature ILC subset are dependent on canonical IL‐7 signaling for development and function, with expression of the receptor (IL‐7Rα) and consumption of IL‐7 dramatically greater in ILCs than their T cell counterparts [24]. Continual differentiation of IL‐7Rα^+^PLZF^+^IL‐18Rα^+^ ILCP into distinct ILC subsets is under the control of a series of lineage‐defining master transcription factors, with all terminal subsets lacking expression of extracellular markers routinely used to identify both lymphoid and myeloid cells, subsequently defining ILCs as lineage negative (Lin^−^) cells (refer to Table 2 for all appropriate lineage markers).

**TABLE 2:** Reagents used for OMIP

Specificity	Fluorochrome	Clone	Purpose
CD45	BUV395	30‐F11	Pan leukocytes
IL‐7Rα	PE‐Cy5	SB/199	Pan ILC
PLZF	PE	MAGS21F7	ILCP
IL‐18Rα	PerCP‐eFluor 710	P3TUNYA	ILCP and ILC1
T‐bet	BV421	4B10	ILC1 and ILC3 subsets
NKp46	PE/Dazzle 594	29A1.4	ILC1, ILC3 subsets and NK cells
CD49a	BUV496	Ha31/8	ILC1 subsets and trNK cells
TRAIL	PE‐Cy7	N2B2	ILC1 subsets
IFN‐γ	BUV737	XMG1.2	ILC1 and NK cells
GATA‐3	BB700	L50‐823	ILC2
KLRG1	BV480	2F1	ILC2
ST2	R718	U29‐93	ILC2
IL‐5	APC	TRFK5	ILC2
IL‐13	eFluor 450	eBio13A	ILC2
RORγt	BV786	Q31‐378	ILC3
CCR6	BV711	140706	ILC3 subsets
IL‐17A	BV650	TC11‐18H10.1	ILC3
IL‐22	Alexa Fluor 647	Poly5164	ILC3
CD49b	eFluor 506	DX5	NK cells
Ki‐67	Alexa Fluor 532	SolA15	Proliferating cells
B220	FITC	RA3‐6B2	B cell subsets (Lin dump)
CD3	FITC	145‐2C11	T cell subsets (Lin dump)
CD4	FITC	GK1.5	T cell subsets (Lin dump)
CD5	FITC	53‐7.3	T cell and B cell subsets (Lin dump)
CD11b	FITC	M1/70	Myeloid/NK/Granulocytes (Lin dump)
CD11c	FITC	N418	Myeloid cells/Granulocytes (Lin dump)
CD19	FITC	1D3/CD19	B cell subsets (Lin dump)
F4/80	FITC	BM8	Macrophages (Lin dump)
FcεR1α	FITC	MAR‐1	Mast cells and Basophils (Lin dump)
Gr‐1	FITC	RB6‐85C	Myeloid cells/Granulocytes (Lin dump)
TCRβ	FITC	H57‐597	αβ T‐cells (Lin dump)
TCRγδ	FITC	UC7‐13D5	γδ T‐cells (Lin dump)
Ter119	FITC	TER‐119	Red blood cells (Lin dump)
Dead cells	LIVE/DEAD™ Blue	‐	Viable cells

ILC1s are characterized by their constitutive expression of T‐bet (encoded by *Tbx21*), which is central for their production of interferon (IFN)‐γ and ensuing response to intracellular pathogens following IL‐12 and IL‐18 stimulation [18, 25]. Owing to their similarities with cytotoxic NK cells, ILC1s express NKp46, however tissue‐specific distinctions can be made between these two subsets via the preferential expression of CD49a and/or TRAIL by ILC1, in conjunction with the absence of CD11b and CD49b [3, 18, 25].

ILC2s represent the most abundant subset of ILCs in mouse lungs [5] and are dependent on the expression of GATA‐3 for maintenance and survival [26, 27]. Analogous to their Th2 counterparts, ILC2s play a key role in controlling helminth infection [26, 28, 29], while perpetuating allergen‐induced allergic inflammation [30, 31, 32, 33] via the production of IL‐5 and IL‐13 in response to alarmins IL‐33, IL‐25, and thymic stromal lymphopoietin (TSLP). Given their extensive roles in both immunity and inflammation, ILC2s are known to display dynamic expression of extracellular markers associated with pro‐inflammatory functions, exemplified via variations in IL‐33 receptor (ST2) and KLRG1 expression depending on the inflammatory signal initiating the primary response [13, 34, 35]. Moreover, the inflammation‐dependent plasticity of ILC2s has been demonstrated in both mouse lungs and human peripheral blood, whereby stimulation with IL‐1β, IL‐12, and IL‐18 promotes ILC2s to adopt an ILC1‐like transcriptional and functional profile associated with the expression of T‐bet (*Tbx21*) and production of IFN‐γ [5, 6, 34, 36]. Similarly, human ILC2s, in response to IL‐1β and IL‐23 stimulation, transdifferentiate into a subset exhibiting ILC3‐like characteristics, evidenced by upregulation of the ILC3 signature transcription factor RORγt and production of IL‐17 [8, 37].

ILC3s are a heterogeneous subset and contribute broad roles in combating against extracellular microbes, including fungi and bacteria, via the production of IL‐17A and IL‐22 following IL‐1β and IL‐23‐mediated activation [38, 39, 40]. Representing the dominant ILC subset within the steady state siLP [41], ILC3s are strictly reliant upon RORγt (encoded by *Rorc*) expression for development and function [39, 42, 43]. However, mouse ILC3s can be further classified into three subtypes based on the differential expression of NKp46 and CCR6; namely NKp46^+^CCR6^−^ (NCR^+^), NKp46^−^CCR6^−^ (NCR^−^), and NKp46^−^CCR6^+^ (CCR6^+^ LTi‐like) ILC3s [42, 44, 45]. Moreover, NCR^+^ ILC3s share transcriptional expression of T‐bet with ILC1s, crucial for their expression of NKp46 and endowing them with the ability to produce IFN‐γ [46, 47]. Furthermore, stimulation of mouse tissue‐specific NCR^+^ ILC3s with IL‐12 promotes downregulation of RORγt and concomitant upregulation of T‐bet, further promoting a phenotype associated with IFN‐γ^+^ ILC1s [4] and demonstrating their plasticity in response to environmental cues.

**FIGURE 1:**
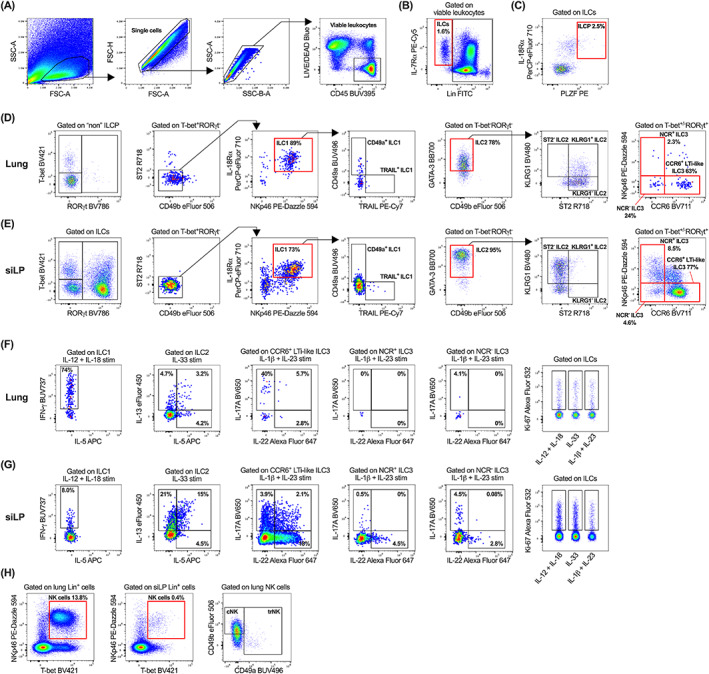
Gating strategy to characterize innate lymphoid cell (ILC) populations and their subset‐specific cytokine and proliferative profiles. After exclusion of cellular debris, doublets, red blood cell contamination and non‐viable CD45^+/−^ cells, viable CD45^+^ leukocytes (A) were gated on IL‐7Rα and lineage (CD3, CD4, CD5, CD11b, CD11c, CD19, B220, F4/80, FcεR1, Gr1, TCRβ, TCRγδ, Ter119; Lin) negative cells to identify ILCs (B). ILCPs were then identified as IL‐18Rα^+^PLZF^+^ cells (C). Characterization of ILC1, ILC2, NCR^+^, NCR^−^ and CCR6^+^ LTi‐like ILC3 subsets in the lung (D) and siLP (E). All samples in panel (A–E) were ex vivo stimulated for 5 h with PMA/Ionomycin/BFA. Single cell suspensions were ex vivo stimulated for 5 h with PMA/Ionomycin/BFA and either IL‐12 and IL‐18 (pro‐ILC1), IL‐33 (pro‐ILC2), or IL‐1β and IL‐23 (pro‐ILC3) and ILC subsets were assessed for production of subset‐specific cytokines (IFN‐γ, IL‐5, IL‐13, IL‐17A, and IL‐22) and cellular proliferation (Ki‐67) within the lung (F) and siLP (G). (H) Identification of NK cells within the lung and siLP, with downstream characterization of lung tissue‐resident NK (trNK) and circulating NK (cNK) cells. Plots in panel (A–C) are representative of individual lung samples. For panels (B–H), viable leukocytes from three technical lung or siLP replicates were concatenated into single FCS files prior to gating. All fluorochrome‐conjugated antibodies were titrated (Figure S1) during panel optimization and manual gating was determined using fluorescence minus one controls (Figure S2) where necessary [Color figure can be viewed at wileyonlinelibrary.com]

Given this microenvironmental‐driven impact on ILC heterogeneity, the developmental phase of this full spectrum flow cytometry panel involved the prioritization of definitive ILCP, ILC1, ILC2, and ILC3 transcription factors, as reflected in the gating strategy (Figure 1). An added benefit of full spectrum flow cytometry is the ability to remove red blood cell contamination by plotting side‐scatter (SSC) captured by the 405 nm violet laser against SSC‐B from the 488 nm blue laser [48] (Figure 1A), thus ensuring purity of downstream terminal cell populations. Viable leukocytes (LIVE/DEAD Blue^−^ CD45^+^; Figure 1A) were selected and total ILCs were defined by their expression of IL‐7Rα^+^ and lack of lineage markers used to routinely define T cells, B cells, myeloid cells, and red blood cells (CD3, CD4, CD5, CD11b, CD11c, CD19, B220, F4/80, FcεR1, Gr1, TCRβ, TCRγδ, Ter119; Figure 1B). ILCPs within the lung were characterized by their ubiquitous expression of PLZF, in combination with IL‐18Rα^+^ (Figure 1C). Mature ILC subsets within the lung (Figure 1D) and siLP (Figure 1E) were stratified as T‐bet^+^RORγt^−^ST2^−^CD49b^−^IL‐18Rα^+^NKp46^+^CD49a^+/−^TRAIL^+/−^ ILC1s, GATA‐3^+^T‐bet^−^RORγt^−^KLRG1^+/−^ST2^+/−^ ILC2s and either RORγt^+^T‐bet^+/−^NKp46^+^CCR6^−^ (NCR^+^), RORγt^+^T‐bet^+/−^NKp46^−^CCR6^−^ (NCR^−^) or RORγt^+^T‐bet^+/−^NKp46^−^CCR6^+^ (CCR6^+^ LTi‐like) ILC3s. Regarding ILC2s, it is important to note that ST2 expression on ex vivo stimulated siLP ILC2s is largely absent (Figure 1E) [9, 49]. Moreover, while lung ILCs lack CD4 expression [50], intestinal CCR6^+^ LTi‐like ILC3s can be sub‐classified as CD4^+^ and CD4^−^ [51]. As such, the inclusion of CD4 within the lineage cocktail may influence the characterization of ILC3 subsets on a tissue‐specific basis. ILC cytokine production and proliferation within the lung (Figure 1F) and siLP (Figure 1G) was assessed in response to subset‐specific activation signals via ex vivo stimulation with phorbol 12‐myrisate 13‐acetate (PMA), Ionomycin and Brefeldin A, and either IL‐12 and IL‐18 (pro‐ILC1), IL‐33 (pro‐ILC2) or IL‐1β and IL‐23 (pro‐ILC3). Stimulation with ILC subset‐specific cytokines clearly demonstrates a heightened propensity for IL‐13 and IL‐22 production by siLP ILC2s and ILC3 subsets respectively, whereas lung ILC1s significantly upregulate IFN‐γ compared to their siLP counterparts. Although the focus of this panel was to accurately define ILCPs and ILC1‐3 subsets, inclusion of CD11b within the lineage cocktail enables the enumeration of conventional mature CD11b^+^NKp46^+^T‐bet^+^ NK cells (Figure 1H) [52], with additional characterization of lung tissue‐resident (trNK) and circulating (cNK) populations on the basis of CD49a and CD49b expression [53] (Figure 1H). Markers including KLRG1 [54] and RORγt [55], and cytokines IFN‐γ, IL‐5, IL‐13 [56] and IL‐22 [57] are all reportedly expressed by NK cell subsets based on maturation, tissue localization and disease state, further highlighting the diversity of this OMIP.

In summary, we present here the first full spectrum flow cytometry panel (Table 2) to comprehensively profile the phenotypic and functional state of ILCs in multiple mouse tissues. Moreover, by maintaining availability on the 355 nm ultraviolet laser, this panel can be expanded to incorporate additional markers of interest with little impact on the overall Complexity™ Index (a value of how distinct a collection of spectral signatures are from one other when unmixed simultaneously), thereby providing a valuable resource within the rapidly expanding field of ILC biology.

## AUTHOR CONTRIBUTIONS

**Kyle T Mincham:** Conceptualization (lead); data curation (lead); formal analysis (lead); investigation (lead); methodology (lead); validation (lead); visualization (lead); writing – original draft (lead); writing – review and editing (equal). **Robert J Snelgrove:** Conceptualization (supporting); funding acquisition (lead); project administration (lead); resources (lead); supervision (lead); writing – original draft (supporting); writing – review and editing (equal).

## CONFLICT OF INTEREST

The author declare that no conflicts of interest exist.

3

### PEER REVIEW

The peer review history for this article is available at https://publons.com/publon/10.1002/cyto.a.24702.

## ETHICS STATEMENT

All mouse experiments were performed in accordance with the recommendations in the Guide for the Use of Laboratory Animals of Imperial College London, with the ARRIVE (Animal Research: Reporting of In Vivo Experiments) guidelines. All animal procedures and care conformed strictly to the UK Home Office Guidelines under the Animals (Scientific Procedures) Act 1986, and the protocols were approved by the Home Office of Great Britain.

## Supporting information


**APPENDIX S1.** Supporting Information.

**MIFlowCyt** Mi Flow checklist.
