# Supplementary material for: OMIP‐086: Full spectrum flow cytometry for high‐dimensional immunophenotyping of mouse innate lymphoid cells
Source: Cytometry A. 2022 Nov 4;103(2):110–6. doi: 10.1002/cyto.a.24702 (PMC10953369; doi:10.1002/cyto.a.24702)
Supplement: Supplementary file 2 — MIFlowCyt Mi Flow checklist. [file CYTO-103-110-s002.docx]

**Cytometry Part A**

**Author Checklist: MIFlowCyt-Compliant Items**

| **Requirement** | **Please Include Requested Information** |
| --- | --- |
| 1.1. Purpose | To investigate heterogeneity and overlap of previously described autoimmune prone B cell subsets in systemic sclerosis and healthy controls |
| 1.2. Keywords | CD21lo B cells  Systemic sclerosis  Interstitial lung disease |
| 1.3. Experiment variables | None. All samples were analyzed ex vivo without additional manipulation. |
| 1.4. Organization name and address | Samples were acquired at:  Vanderbilt University Medical Center  Nashville, TN 37232 |
| 1.5. Primary contact name and email address | Peggy L. Kendall  peggy.kendall@wustl.edu |
| 1.6. Date or time period of experiment | Samples were collected from 9/2017-2/2020. Samples were acquired in 12/2017 and 5/2022. Additional data analysis occurred from 12/2017-8/2021 and 6/2022-7/2022. |
| 1.7. Conclusions | Significant overlap exists between previously described autoimmune-prone B cell subsets. |
| 1.8. Quality control measures | 5 healthy controls and 11 systemic sclerosis patients were used for this study in the initial cohort. The Expansion Cohort included an additional 9 systemic sclerosis patients and 11 healthy controls. |
| 2.1.1.1. (2.1.2.1., 2.1.3.1.) Sample description | Peripheral blood |
| 2.1.1.2. Biological sample source description | Peripheral blood mononuclear cells isolated by CPT tubes |
| 2.1.1.3. Biological sample source organism description | Homo sapiens |
| 2.1.2.2. Environmental sample location | United States |
| 2.3. Sample treatment description | Cryopreserved PBMCs were thawed. No *in vitro* manipulation occurred prior to experimentation |
| 2.4. Fluorescence reagent(s) description | CD19-BUV395 (SJ25C1), CD5-BV785 (UCHT2), and CD21-PE (B-ly4) from BD Biosciences and IgD-AF488 (IA6-2), IgM-PacBlue (MHM-88), CD27-PEDazzle (O323), CD38-AF700 (HIT2), CD24-PECy7 (ML5), CD10-PECy5 (HI10a), CD3-BV510 (OKT3), CD14-BV510 (M5E2), and CD16-BV510 (3G8) from BioLegend |
| 3.1. Instrument manufacturer | BD Biosciences |
| 3.2.1 Instrument model | LSR II (Initial Cohort) |
| 3.2.2 Instrument model | LSR Fortessa (Expansion Cohort) |
| 3.3.1 Instrument configuration and settings  (LSR II) | \| Laser \| Channel \| Long Pass \| Short Pass \| \| --- \| --- \| --- \| --- \| \| Red laser (635nm) \| APC-Cy7 \| 735LP \| 780/60 \| \|  \| APC \|  \| 670/14 \| \|  \| Alexa Fluor 700 \| 685LP \| 730/45 \| \| Blue laser (488nm) \| FITC \| 505LP \| 530/30 \| \|  \| SSC \|  \| 488/10 \| \| Green laser (545nm) \| PE-Dazzle/Tx-Red \| 600LP \| 610/20 \| \|  \| PE-Cy7 \| 735LP \| 780/60 \| \|  \| PE-Cy5 \| 635LP \| 675/20 \| \|  \| PE \|  \| 575/26 \| \| Violet laser (405nm) \| BV785  (read on Qdot 705) \| 685LP \| 780/60 \| \|  \| BV510/AmCyan \| 505LP \| 515/20 \| \|  \| Pacific Blue \|  \| 450/50 \| \| UV laser (355nm) \| BUV395/DAPI \|  \| 450/50 \| |
| 3.3.2 Instrument configuration and settings  (LSR Fortessa) | \| Laser \| Channel \| Long Pass \| Short Pass \| \| --- \| --- \| --- \| --- \| \| Red laser (635nm) \| APC-Cy7 \| 750LP \| 780/60 \| \|  \| APC \|  \| 670/14 \| \|  \| Alexa Fluor 700 \| 690LP \| 790/65 \| \| Blue laser (488nm) \| FITC \| 505LP \| 525/50 \| \|  \| SSC \|  \| 488/10 \| \| Green laser (545nm) \| PE-Dazzle/Tx-Red \| 595LP \| 610/20 \| \|  \| PE-Cy7 \| 750LP \| 780/60 \| \|  \| PE-Cy5 \| 635LP \| 670/30 \| \|  \| PE \|  \| 582/15 \| \| Violet laser (405nm) \| BV785  (read on Qdot 705) \| 685LP \| 780/60 \| \|  \| BV510/AmCyan \| 505LP \| 525/50 \| \|  \| Pacific Blue \|  \| 450/40 \| \| UV laser (355nm) \| BUV396 \|  \| 379/28 \| |
| 4.1. List-mode data files | *We recommend all authors to submit their data files to [http://flowrepository.org](http://flowrepository.org/) and to make them available for the peer-review process. If you have done so, please let us know by inserting the following codes (replace the red text):  All datasets are publicly available at the time of resubmission.  Initial Cohort: https://flowrepository.org/id/FR-FCM-Z5KF  Expansion Cohort: https://flowrepository.org/id/FR-FCM-Z5KE |
| 4.2. Compensation description | Single color compensation was performed using peripheral blood mononuclear cells at the time of sample acquisition. Software compensation was performed using BD FACSDiva^TM^. |
| 4.3. Data transformation details | For biaxial gating, FCS files were analyzed using FlowJo v10.4 software (Tree Star Inc.). For tSNE analysis, FCS files were analyzed using Cytobank software. Data were transformed to arcsinh scales with varying cofactors ranging from 150 to 6,000. FCS files from all healthy donors were concatenated using the FCS file concatenation tool provided by Cytobank. |
| 4.4.1. Gate description | B cells: CD19+CD3-CD14-CD16-  CD21lo B cells: CD19+CD3-CD14-CD16-CD27-CD10-CD21-  CD24hiCD38hi: CD19+CD3-CD14-CD16-CD24hiCD38hi  B_ND_ cells: CD19+CD3-CD14-CD16-CD27-IgD+IgM-  DN B cells: CD19+CD3-CD14-CD16-CD27-IgD- |
| 4.4.2. Gate statistics | N/A |
| 4.4.3. Gate boundaries | Primary B cell gating    Gating of autoreactive-prone B cell subsets from B cells is shown in Figure 1 (reproduced below).  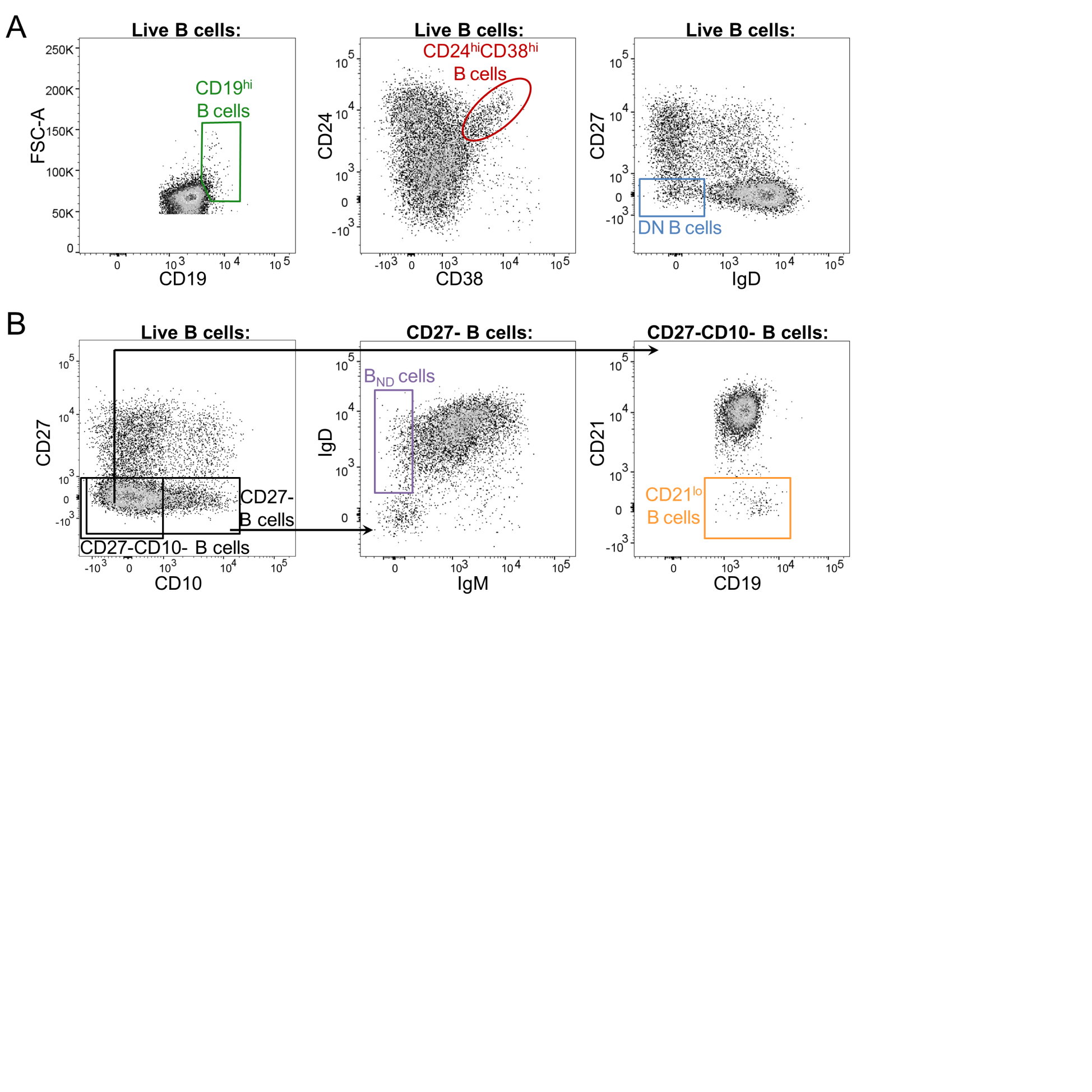 |
